# The ubiquity of frequency effects in first language acquisition*

**DOI:** 10.1017/S030500091400049X

**Published:** 2015-03

**Authors:** BEN AMBRIDGE, EVAN KIDD, CAROLINE F. ROWLAND, ANNA L. THEAKSTON

**Affiliations:** University of LiverpoolESRC International Centre for Language and Communicative Development (LuCiD); Australian National UniversityARC Centre of Excellence for the Dynamics of Language ESRC International Centre for Language and Communicative Development (LuCiD); University of LiverpoolESRC International Centre for Language and Communicative Development (LuCiD); University of ManchesterESRC International Centre for Language and Communicative Development (LuCiD)

## Abstract

This review article presents evidence for the claim that frequency effects are pervasive in children's first language acquisition, and hence constitute a phenomenon that any successful account must explain. The article is organized around four key domains of research: children's acquisition of single words, inflectional morphology, simple syntactic constructions, and more advanced constructions. In presenting this evidence, we develop five theses. (i) There exist different types of frequency effect, from effects at the level of concrete lexical strings to effects at the level of abstract cues to thematic-role assignment, as well as effects of both token and type, and absolute and relative, frequency. High-frequency forms are (ii) early acquired and (iii) prevent errors in contexts where they are the target, but also (iv) cause errors in contexts in which a competing lower-frequency form is the target. (v) Frequency effects interact with other factors (e.g. serial position, utterance length), and the patterning of these interactions is generally informative with regard to the nature of the learning mechanism. We conclude by arguing that any successful account of language acquisition, from whatever theoretical standpoint, must be frequency sensitive to the extent that it can explain the effects documented in this review, and outline some types of account that do and do not meet this criterion.

## INTRODUCTION

Frequency effects are ubiquitous in virtually every domain of human cognition and behaviour, from the perception of facial attractiveness (Grammer & Thornhill, 1994) and the processing of musical structure (Temperley, 2007) to language change (Bybee, 2010) and adult sentence processing (Ellis, 2002). Our goal in this target article is to argue that frequency effects are ubiquitous also in children's first language acquisition, and to summarize the different types of frequency effect that are observed across all of its subdomains. We argue, very simply, that frequency effects constitute a phenomenon for which any successful theory must account. Such a theory might be a generativist/nativist account, under which children have innate knowledge of abstract categories, but are sensitive to the frequency with which exemplars of these categories are present in the input (e.g. see Yang, 2004, for a review). It could equally be a constructivist/usage-based account, under which children build up abstract constructions on the basis of the input, with the aid of little or no innate linguistic knowledge (e.g. Tomasello, 2003). Regardless of whatever other theoretical assumptions are made, any successful account of language acquisition will need to incorporate frequency-sensitive learning mechanisms.

It is important, at the outset, to clarify our claim. We do not argue that sensitivity to input frequency must be the defining feature, or even the most important feature, of a successful account of acquisition (i.e. we do not argue for a frequency-driven or frequency-based mechanism). It is not difficult to think of factors that are more important than input frequency in at least some scenarios. For example, if we consider the straightforward token frequency of lexical items, there is every reason to believe that children will make more effort to store low-frequency input strings that can be used to obtain desired objects (e.g. *cake*) than higher-frequency strings that cannot (e.g. *the*). We argue, instead, for a learning mechanism that is minimally frequency sensitive, under which input frequency need not be the chief determinant of acquisition in all cases.

It is also important to make clear that a frequency-sensitive learning mechanism need not (and most probably does not) entail a mechanism that “computes and matches the frequency of various elements in the input” or acquires “knowledge of frequency” (Bohnacker, 2007, pp. 54–55; see Ambridge, 2010, for discussion). Frequency in this sense (i.e. token frequency) need not be represented per se, but may be instantiated in the strength of representations or neural connections in exactly the same way that explicit and implicit memory for stimuli of all types is boosted by repetition. Similarly, type frequency information may be represented only indirectly, instantiated in the similarity structure of stored exemplars.

Thus far, our claim is relatively uncontroversial: few would disagree that at least some domains of language acquisition show frequency effects at some level (though see Roeper, 2007). But our claim is much broader: we propose that frequency effects are ubiquitous in every domain of child language acquisition and that any apparent null finding simply reflects a failure to conceptualize frequency appropriately, to find a sufficiently sensitive dependent measure, or to hold constant other relevant factors.

We illustrate this claim with evidence from four core domains: the acquisition of single words, inflectional morphology, simple syntactic constructions, and more advanced constructions. Within these sections, our overarching claim takes the form of five inter-related theses:

1. *Levels and Kinds Thesis*. Frequency effects exist at all levels and are of many different kinds. They are observed not only at the level of concrete lexical strings (perhaps the prototypical frequency effect), but also at the level of abstract categories (e.g. particular orderings of SUBJECT and OBJECT) and cues (e.g. animacy, givenness). There are token frequency effects (e.g. at the level of the word, the more often you hear a word, the more likely you are to learn it) and type frequency effects (e.g. at the level of inflectional morphology, the more verbs you hear with a particular inflectional ending, the more likely you are to learn that ending). There are effects of absolute frequency (e.g. high-frequency words will be learned earlier than low-frequency words) and relative frequency (e.g. of two competing forms, the most frequent will be dominant).
2. *Age of Acquisition (AoA) Thesis*. All other things being equal, frequent forms will be acquired before less-frequent forms. As we will see in more detail, since all other things are rarely – if ever – equal, this claim does not entail a one-to-one relationship between frequency and age of acquisition (and neither is the definition of ‘acquisition’ straightforward).
3. *Prevent Error Thesis*. High-frequency forms prevent (or at least reduce) errors in contexts in which they are the target. For example, we will see that third person singular verb forms – almost always the most frequent in the input – are invariably produced correctly in third person singular contexts.
4. *Cause Error Thesis*. Conversely, high-frequency forms also cause error in contexts in which a competing, related lower-frequency form is the target. For example, we will see that high-frequency third person singular verb forms are often used inappropriately in third person plural contexts.
5. *Interaction Thesis*. Finally, we propose that frequency effects will interact with other effects. One example is utterance position: high-frequency verbs are generally learned before lower-frequency verbs (a main effect of verb frequency), and this effect is boosted for verbs that occur frequently in utterance-final position (an interaction of verb frequency by utterance position). The downside of these interactions is that they can make frequency effects difficult to detect. The upside is that these interactions are generally informative with regard to the other factors that we need to build into the learning mechanism (e.g. sensitivity to utterance position or temporal ordering).

The remainder of this article synthesizes the considerable empirical support that exists for each of our theses across four domains: single words, inflectional morphology, simple syntactic constructions, and more advanced constructions. This strategy inevitably entails a degree of repetition and overlap, for which we make no apology. The point is that the frequency effects captured by these five theses do not rely on cherry-picking particular domains or debates, but are ubiquitous across first language acquisition.

At this point, we should also clarify that whenever we refer to frequency in this article, we mean input frequency. It is likely that children also show effects of output frequency (e.g. better performance with strings that they produce more often). However, we do not discuss such effects, as, other than in the domain of phonology (e.g. DePaolis, Vihman & Keren-Portnoy, 2011), few studies have attempted to dissociate effects of input and output frequency. Indeed, this will often prove to be rather difficult, given that the frequency distributions of utterances produced by children and their caregivers are generally extremely similar.

## SINGLE WORDS

This section presents evidence for perhaps our two most straightforward theses; that – all else being equal – frequent forms are (a) acquired earlier than less frequent ones (AoA Thesis) and (b) associated with lower rates of error, and higher rates of correct use (Prevent Error Thesis). The findings discussed also constitute evidence for our Interaction Thesis.

In the adult psycholinguistics literature, frequency effects at the single-word level have been almost universally accepted for over a hundred years (e.g. Ebbinghaus, 1913 [1885]; though for one dissenting view, see Roeper, 2007, p. 26). Higher-frequency words are (i) remembered more easily in both recall and recognition tasks (e.g. Hulme, Roodenrys, Schweickert, Brown, Martin & Stuart, 1997), (ii) more easily identified, including when subject to audio degradation (Howes, 1957; Savin, 1963; Luce, 1986), (iii) mispronounced less often (Dell, 1990), (iv) judged more quickly and accurately in lexical decision tasks (Forster, 1976; Balota, Cortese, Sergent-Marshall, Spieler & Yap, 2004; Brysbaert & New, 2009), and (v) correctly judged as high-frequency in subjective frequency-estimation tasks (Balota, Pilotti & Cortese, 2001).

Similar frequency effects are apparent in children's acquisition (our AoA Thesis). As a rule, children learn frequent words before infrequent ones: American English-speaking children's most common first words in production are (in order) *Daddy, Mommy, bye, hi, uh-oh, dog, no, ball, baby*, and *book* (Fenson *et al.*, 1994), not, for example, *coffee* and *computer* (words that children certainly hear, just less frequently).

However, there is an important caveat to be made here, one that has sometimes been misunderstood. Our claim is not that frequency is the only predictor, but that frequent words are learned before infrequent ones, all other things being equal. Thus, we do not predict that there will be a one-to-one relationship between frequency and age of acquisition (which is just as well, since children's first word is rarely *the*). There are many other factors that influence acquisition: a word is more likely to be early learned if it is, *inter alia*, relevant to the child's communicative goals (Ninio, 2006), associated with an easily identifiable referent (Gentner, 1982), imageable (Bird, Franklin & Howard, 2001), aligned with prosodic boundaries (Christophe & Dupoux, 1996), easy to segment from the continuous speech stream (Monaghan & Christiansen, 2010), easy to say (Vihman & Vihman, 2011), and attested in a wide range of contexts (Naigles & Hoff-Ginsberg, 1998; Küntay & Slobin, 2002). Our prediction, thus, is that, in a regression analysis, input frequency will make a significant unique contribution to the variance of the outcome measure (in this case, age of acquisition), even when all of these other factors are included in the model. Although few, if any, studies have controlled for all of these factors, this prediction is, in general, very well supported. For example, independent effects of input frequency on age of acquisition have been found looking across verbs (Naigles & Hoff-Ginsberg, 1998; Smiley & Huttenlocher, 1995; Theakston, Lieven, Pine & Rowland, 2004), adjectives (Blackwell, 2005), and nouns and function words (Goodman, Dale & Li, 2008).

Turning now to our Prevent Error Thesis, the domain of single-word acquisition provides ample evidence that high-frequency forms are associated with lower rates of error, and higher rates of correct production and comprehension, than lower-frequency forms. The most direct evidence comes from studies in which word frequency is manipulated experimentally, which allow researchers to control out confounding factors using counterbalancing procedures. For example, Schwartz and Terrel (1983) taught one- to three-year-old children either four novel nouns or four novel verbs. Each individual word+object/action pair was presented with high frequency (a total of 20 presentations) for half of the children and low frequency (10 presentations) for the remainder. Thus their finding that the high-frequency words were correctly recalled significantly more often than low-frequency words (a finding that held for both nouns and verbs) cannot realistically be attributed to any factor other than input frequency (for similar studies with L2 learners and children with SLI, see Rice, Oetting, Marquis, Bode & Pae, 1994; Wang & Koda, 2005; McGregor, Sheng & Ball, 2007; Joe, 2010; Eckerth & Tavakoli, 2012).

At the same time, while it is useful to be able to control factors such as imageability, prosody, and utterance position experimentally, our Interaction Thesis holds that interactions between frequency and one or more of these other effects are informative with regard to the nature of the language learning mechanism. A detailed analysis of all of these potential interactions is beyond the scope of the present article. However, two findings are relevant as an illustration of the informative nature of interactions between frequency and a second factor, here utterance position and utterance length. In their study of verb acquisition, Naigles and Hoff-Ginsberg (1998) found that, in addition to overall input frequency, input frequency in utterance-final position was a significant predictor of age of acquisition. Relatedly, Brent and Siskind (2001) found that age of acquisition was best predicted not by a word's overall input frequency, but by the frequency with which it appeared as the sole constituent of an utterance.

Consequently, interactions with other factors are not merely a source of noise that must be eliminated in order to observe frequency effects or that can be appealed to in order to explain away null findings. Rather, these interactions can constrain our theories, by informing us about the nature of the learning mechanism, For example, the finding of an interaction between frequency and utterance position (e.g. Naigles & Hoff-Ginsberg, 1998) suggests that we need to posit a learning mechanism that is sensitive to temporal order, rather than, for example, a mechanism that processes entire input sequences one batch at a time. Thus, our Interaction Thesis allows us to make general predictions about the learning mechanism that can be tested in other domains (e.g. morphosyntax; e.g. Freudenthal, Pine, Aguado-Orea & Gobet, 2007), and perhaps even non-linguistic domains such as memory for musical notes or sequences (e.g. Berz, 1995).

## INFLECTED FORMS

In this section we consider children's acquisition of morphologically inflected forms (mainly verbs, but also nouns), and the evidence that this domain provides for three of our theses. The first is that high-frequency forms (in this case surface strings) are associated with lower rates of error, and higher rates of correct use (Prevent Error Thesis). The second is that high-frequency forms can cause errors when used in inappropriate contexts, which – in this domain – essentially means inappropriate person/number contexts (Cause Error Thesis). The third is that there are different types of frequency effect (Levels & Kinds Thesis); the specific kinds of error contrasted here being (a) relative versus absolute and (b) type versus token frequency effects.

Many early investigations concluded that no effect of input frequency could be observed in the domain of the acquisition of inflectional morphology. For example, looking across fourteen different morphemes, Brown (1973) found no correlation between input frequency and age of acquisition, whether looking at individual child–caregiver dyads or across the whole group (see also Newport, Gleitman & Gleitman, 1977; Gleitman & Wanner, 1984; De Villiers, 1985; though see Moerk, 1980, for a reanalysis of Brown's data that did yield frequency effects, and Moerk, 1981, and Pinker, 1981, for further discussion).

The problem with this study, however, is the use of age of ‘acquisition’ (which usually entails first production) in naturalistic speech as the dependent measure. This measure is problematic because children are motivated to talk about certain topics at the expense of others, and thus have little occasion to produce certain inflected forms, even if they know them well. For example, despite their high frequency in the input, children rarely produce second person singular forms. Raw production data simply cannot tell us whether children (a) have failed to learn these forms despite their high frequency or (b) have learned these forms, but find little use for them (e.g. young children are not interested in talking about what their listener is doing).

One solution is to use as our dependent measure not the age at which a particular form is first produced or the raw frequency of these forms in the child's speech but the proportion of correct versus incorrect uses in obligatory contexts. Because this is a proportional measure, it controls for the confound that, for example, first person singular contexts far outnumber third person singular contexts in children's speech. Thus, a better way of examining frequency effects is to test the prediction that the higher the frequency of the individual word form (i.e. the inflected, realized form, as opposed to the lemma), the higher the rate (i.e. proportion) of correct use, and the lower the rate of errors; whether errors of commission or omission (our Prevent Error Thesis).

When this prediction is tested, clear effects of input frequency are found, in both naturalistic (e.g. Theakston, Lieven, Pine & Rowland, 2005; Theakston & Lieven, 2005, 2008; Theakston & Rowland, 2009) and experimental studies (e.g. Leonard, Caselli & Devescovi, 2002; Dabrowska & Szczerbinski, 2006; Räsänen, Ambridge & Pine, 2014). For example, Dabrowska and Szczerbinkski (2006) found a correlation between the input frequency of genitive, dative, and accusative Polish noun case-marking inflections, and children's correct performance with novel noun inflection. These frequency effects are not merely an artefact caused by children's memory or processing difficulties. In adult studies of production latency, differences are found between more and less frequent forms of the same lemma (e.g. *playing* vs. *plays*; Jescheniak & Levelt, 1994). Though, again, it is important to bear in mind that – consistent with our Interaction Thesis – frequency interacts with other factors, including serial position (e.g. Freudenthal *et al.*, 2007; Gagarina, 2007; Freudenthal, Pine & Gobet, 2010;) and the form most recently produced by an interlocutor (e.g. Krajewski, Theakston, Lieven & Tomasello, 2011).

A number of findings from this domain illustrate another of our theses: high-frequency forms not only prevent errors in contexts where they are the target, but Cause Error where a lower-frequency form is the target. For example, in a naturalistic study of child Spanish, Aguado-Orea (2004) found high error rates for third person plural target forms (which are very rare in the input), almost all of which involved the substitution of much more frequent third person singular forms (see also Räsänen, Ambridge & Pine, unpublished observations, for Finnish). Similar findings were reported by Dabrowska (2008) for case-marking errors, Theakston and Rowland (2009) for auxiliary *is*-for-*are* errors, and Cameron-Faulkner and Kidd (2007) for *are*-for-*am* errors (e.g. *I are playing).

Turning now to our Levels and Kinds Thesis, the domain of inflectional morphology also provides a useful illustration of the difference between the effects of token and type frequency. Token frequency is simply the number of times that a particular string (e.g. *Mummy*) occurs in the child's input. Type frequency is the number of different items that follow a particular morphosyntactic pattern. Precisely what is meant by the term ‘following a particular pattern’ varies from domain to domain, but a reasonably straightforward case occurs in the English past tense system (e.g. Bybee & Slobin, 1982; Bybee & Moder, 1983). For example, the *ow*→*ew* pattern has a high type frequency because many verbs form their past tense in this way (e.g. *blow/blew, know/knew, grow/grew, throw/threw*), whilst the pattern exemplified by *make/made* has a very low type frequency (probably a type frequency of 1).

There is some evidence to suggest that patterns with high type frequency are more productive (i.e. more open to newcomers), though it is often difficult, when considering morphological systems, to separate the effect of type frequency from phonological heterogeneity (Janda, 1990; Forrester & Plunkett, 1994; Bybee, 1995; Hare, Elman & Daughterty, 1995; Plunkett & Nakisa, 1997; Bowerman & Choi, 2001; Dąbrowska & Szczerbinski, 2006; Nicoladis, Palmer & Marentette, 2007; Barðdal, 2008; Suttle & Goldberg, 2011; Kirjavainen, Nikolaev & Kidd, 2012; Ambridge & Lieven, 2014). However, there is also evidence to suggest that inflected forms with very high token frequency (e.g. *said*) constitute unanalyzed frozen phrases, and so do not contribute to analogical generalization at all (e.g. the existence of *say*→*said* does not lead children to produce errors such as *play*→*pled or *obey*→*obed); see Baayen and Lieber (1991), Bybee (1995), and Wang and Derwing (1994).

The domain of inflectional morphology, in particular, English verb past tense and noun plural marking, also illustrates a further contrast within our Levels and Kinds Thesis – absolute vs. relative frequency. With regard to absolute frequency, this domain illustrates the common finding that the more frequent the irregular form (in absolute terms), the more likely children are to produce this form, as opposed to an error (also relevant to our Prevent Error Thesis). For example, the high-frequency irregulars *blew* and *feet* are less likely to be over-regularized (e.g. *blowed, *foots) than the low-frequency irregulars *drank* and *shelves* (e.g. *drinked and *shelfs) (Marchman, 1997; Marchman, Wulfeck & Weismer, 1999; Maslen, Theakston, Lieven & Tomasello, 2004).

With regard to relative frequency, errors are particularly common when the target form is infrequent relative to a high-frequency competitor form (e.g. a ‘zero-marked’ form, as in Yesterday I wanted/*want an ice-cream). For example, focusing on zero-marking errors in the domain of noun plural marking, Matthews and Theakston (2006) found that children often produced *two mouse, because the target (*mice*) is less frequent in the input than the competitor (*mouse*), but rarely produced *two foot, because the target (*feet*) is more common in the input than the competitor (*foot*).

The implication of our Levels and Kinds Thesis is that we need an account that incorporates different types of frequency effect: both absolute frequency (e.g. to explain why *Mummy* is learned before *coffee* or why *feet* resists overgeneralization better than does *shelves*) and relative frequency (e.g. to explain why children substitute low-frequency third person plural verb forms with erroneous high-frequency third person singular forms of the same verb, or *mice* with *mouse*, but not *feet* with *foot*). This does not necessarily entail positing that children must ‘decide’ whether to pay attention to absolute or relative frequency in a particular domain (which is just as well, since such a position would be untenable). Children are clearly sensitive to both relative and absolute frequency; the challenge is to posit a learning mechanism that yields effects at both of these levels.

One example is the learning model of Rescorla and Wagner (1972). In this model, the assumption is that a meaning or entity (e.g. MUMMY) has only a certain amount of associative strength to give out. If this entity is paired with one label (e.g. *Mummy*), this associative strength does not need to be shared: every pairing of MUMMY and *Mummy* strengthens the association between the two. If an entity (e.g. MOUSE) is paired with two labels (e.g. *Mouse, Mice*), its associative strength is shared between the two: every pairing of MOUSE and *Mouse* strengthens the link between MOUSE and *Mouse* at the expense of the link between MOUSE and *Mice*, and vice versa (Ramscar, Dye & McCauley, 2013; see Legate & Yang, 2007, for a version of this account in the domain of Optional Infinitive errors). Regardless of the merits or otherwise of an associative account of word learning, the point is simply that a learning mechanism can yield effects of both absolute and relative frequency, without it somehow having to ‘decide’ which to use in each domain.

The moral here is that a sophisticated consideration of different possible types of frequency effect (Levels and Kinds Thesis) allows us to constrain theory building in a way that simplistic correlations between the input and output frequency of particular strings cannot. The need to account for effects of both absolute and relative frequency forces us to posit particular types of acquisition model that we may not otherwise have considered; specifically those that build in some form of competition between words with similar meanings and/or surface forms (MacWhinney, 2004). Thus a ‘frequency effect’ can never be an explanation or answer in its own right. Rather, it poses a question: What type of learning mechanism is needed to yield the particular types of frequency effect observed?

## MULTIWORD STRINGS AND SIMPLE SYNTACTIC CONSTRUCTIONS

This section discusses frequency effects at the levels of multiword strings and grammatical (i.e. sentence-level) constructions. This domain is useful in particular for illustrating our claim that there exist many different types of frequency effect (Levels and Kinds Thesis), as well as providing evidence for our Prevent Error, Cause Error, and AoA Theses.

### Multiword strings

The first type of frequency effect is one that we have discussed already: frequently occurring strings prevent or reduce errors (Prevent Error). This is true not only of single words (including inflected forms) but also of multiword strings. Bannard and Matthews (2008) found that children are better able to repeat four-word sequences found frequently in child-directed speech (CDS) than less-frequent four-word sequences, even when the frequency of the individual items and bigrams was carefully controlled (e.g. comparing *a cup of tea* with *a cup of milk*). Similar findings were observed by Matthews and Bannard (2010), Arnon and Snider (2010), and Arnon and Clark (2011; see also Conklin & Schmitt, 2012, for an overview of such effects in adults). In a different context, a number of studies (Mintz, 2003; Chemla, Mintz, Bernal, and Christophe, 2009; Weisleder & Waxman, 2010; but see Erkelens, 2009; Stumper, Bannard, Lieven & Tomasello, 2011) have demonstrated that children are also sensitive to frequent frames: “ordered pairs of words that frequently co-occur with exactly one word position intervening (occupied by any word)” (Mintz, 2003, p. 93).

The second type of frequency effect is also one that we have encountered previously: high-frequency strings not only prevent error when used correctly, but seem to cause errors when used incorrectly (Cause Error Thesis). For example, in a study of early negation, Cameron-Faulkner, Lieven, and Theakston (2007) reported that early verbal negation was largely ungrammatical (e.g. *no move, no drop it*), and therefore reflected creative use on the part of the child (multiword utterances containing the negator *no* were very rare in the caregiver's speech). However, they argued that these early errors were in fact frequency driven – the child was using the most frequent, functionally generic, and salient single word negator in the input overall (*no*), which he creatively combined with verbs, resulting in a *no+VERB* frame. Later in development this made way for a shift towards the use of *not+VERB* (e.g. *not going there, not open the lid*), which they argued was due to the high frequency of *not* in multiword utterances in the input, although not necessarily in combination with verbs. Finally, the child shifted towards the use of auxiliary forms (e.g. *Don't sit down here, I can't talk*), but this shift was function-dependent (e.g. prohibition, inability) and was closely tied to the frequency of particular AUX+neg forms (e.g. *don't, can't*) to express particular functions in the input.

These complex effects encompassing frequency of both surface forms and communicative functions pose a challenge for researchers. We currently lack a good understanding of whether and how frequency effects change over the course of development, as a consequence of children's increasing semantic and pragmatic knowledge. Computational models provide one means of investigating how far it is possible to get with relatively simple surface-form learning, provided that the model is sensitive to frequency in an appropriate way (e.g. Freudenthal *et al.*, 2007). Incorporating semantic and/or pragmatic coding into these kinds of model (e.g. Chang, Dell & Bock, 2006) would allow researchers to determine what additional benefit this kind of frequency information provides to the learning mechanism, and how closely the corresponding output matches children's language at different stages in development.

### Simple syntactic constructions

In the domain of simple grammatical constructions, we see effects of frequency at a variety of levels and of different kinds; frequency of (a) individual verbs, (b) verb+argument/construction combinations, and (c) abstract cues to word order (Levels and Kinds Thesis). For example, with regard to verb+argument combinations, the order in which children acquire verbs within the transitive and intransitive constructions is predicted by both the overall frequency of the verbs and the frequency of those verbs in those same constructions in the input (Ninio, 1999; Theakston, Lieven, Pine & Rowland, 2004), consistent with our AoA Thesis. Focusing on arguments, children's use of grammatical objects with verbs that can occur both transitively and intransitively mirrors the relative use of the two constructions with those same verbs in the input (Theakston, Lieven, Pine & Rowland, 2001). Similar findings are observed in so-called weird-word order studies (e.g. Akhtar, 1999; Abbot-Smith, Lieven & Tomasello, 2001; Matthews, Lieven, Theakston & Tomasello, 2005, 2007), in which children follow an experimenter's ungrammatical word order for low-frequency and novel verbs (e.g. *Fox bear rammed, Elmo the car gopping*), but correct the use of a high-frequency verb to the word order in which it has frequently been attested in the input (e.g. *Fox pushed bear*). Indeed, a number of grammaticality judgment studies have demonstrated that sensitivity to the frequency of particular verb+argument structure combinations continues into older childhood and adulthood (MacDonald, 1994, 1999; Seidenberg, 1997; Ellis, 2002; Stefanowitsch & Gries, 2003; Theakston, 2004; Stefanowitsch, 2008; Wonnacott, Newport & Tanenhaus, 2008; Ambridge, Pine & Rowland, 2012; Ambridge, Pine, Rowland & Chang, 2012), with high-frequency combinations again protecting children from error (Prevent Errors).

Continuing our illustration of the Levels and Kinds Thesis, there is evidence that children are sensitive not only to the frequency of particular verb+arugment and verb+construction combinations, but also to the frequency of more abstract cues to word order (possibly at different developmental stages). In particular, investigations of children's developing sensitivity to cues such as word order, case marking, and animacy, in their interpretation of the simple transitive NVN construction, typically show that young children are better able to interpret sentences in which multiple cues indicate the same sentence interpretation than those in which only a single cue operates in isolation or cues conflict. This finding, which has been replicated across a number of languages, reflects the higher frequency of sentences with multiple supporting cues in the input (Bates & MacWhinney, 1982; Slobin & Bever, 1982; Dittmar, Abbot-Smith, Lieven & Tomasello, 2008; Goksun, Küntay & Naigles, 2008; Scott & Fisher, 2009; Chan, Lieven & Tomasello, 2009; Ibbotson, Theakston, Lieven & Tomasello, 2011; Candan, Küntay, Yeh, Cheung, Wagner & Naigles, 2012; Matsuo, Kita, Shinya, Wood & Naigles, 2012; though see Lidz, Gleitman & Gleitman, 2004, for counter-arguments, and Goldberg, 2004, for a critique of their approach). Later in development, however, children start to grasp the significance of individual, often rather infrequent, cues (e.g. the need to prioritise case marking over word order in German, reflecting a shift from the influence of highly frequent SVO word order, to less-frequent but highly reliable case marking; Dittmar *et al.*, 2008).

Further illustrating our Levels and Kinds Thesis, the domain of the acquisition of simple constructions exhibits a particularly interesting and well-studied interaction between type and token frequency. Several studies (Goldberg, Casenhiser & Sethuraman, 2004; Casenhiser & Goldberg, 2005; Goldberg, Casenhiser & White, 2007) have found that children show an advantage for learning the meanings of ‘skewed’ constructions where one or two types constitute the lion's share of all constructional tokens, as compared to ‘balanced’ constructions where the tokens are divided more evenly amongst the types. The picture has been complicated by the fact that some studies have found no advantage for either type of distribution (Year & Gordon, 2009), or even an advantage for a more balanced distribution (Siebenborn, Krajewski & Lieven, unpublished observations; see Johnson & Goldberg, unpublished observations, for discussion: online <http://www.princeton.edu/~adele/Princeton_Construction_Site/Publications_files/SkewedInput.pdf>). Whatever the overall pattern, for our present purposes, the important point is that – again – we see a case where careful examination of the different types of frequency effect observed constrains theory development by forcing us to build models that can yield these complex effects; effects that would have been missed entirely by an approach that focused solely on the relationship between the input and output frequency of particular tokens.

Although we have focused in this domain on our Levels and Kinds Thesis, this is not to say that our other theses are not supported here. Work on the development of simple grammatical constructions also illustrates our Cause Error Thesis. Theakston (2012) found that, when producing simple transitive sentences with a discourse-new subject, children as old as five years often produced an underinformative pronoun subject (e.g. *He* rather than *The cat*). That is, children seemed to overgeneralize a particularly frequent transitive sentence subject, *He* (or perhaps even its ‘givenness’ property) into an inappropriate context (one in which the subject is discourse-new). With regard to the Prevent Error Thesis, Rowland and Noble (2010) found that children showed better comprehension of dative sentences containing novel verbs when the recipient was a proper noun (e.g. *I'm blicking **Teddy** the frog*) than a definite determiner phrase (e.g. *I'm blicking **the Teddy** the frog*). Although other factors are no doubt relevant too (e.g. consecutive determiner+noun sequences are confusing), one relevant factor seems to be that 94% of datives in child-directed speech are of the former type. Thus frequency is preventing errors here; but frequency not of individual lexical items or categories, but of cues to thematic role assignment (e.g. ‘being a proper noun’ is a frequently heard cue to recipienthood).

In summary, whilst input frequency effects are straightforwardly (and hence uncontroversially) observed at the levels of individual words or surface strings, effects at the level of sentence constructions are much more evasive. We have argued, however, that frequency effects – token and type, AoA, and preventing and causing error – are no less ubiquitous in this domain than any other. The reason that they often elude discovery is that they tend to be rather abstract: what is relevant is often the frequency not of surface strings but of pairings between concrete lexical items and abstract constructions, of abstract cues to subjecthood, of type:token ratios within a given construction, and so on. Indeed, even when we might be tempted simply to count the number of occurrences of a particular word (e.g. *go*), the appropriate frequency measure – and the one that yields correlations between children's speech and their input (Theakston, Lieven, Pine & Rowland, 2002) – is the frequency of each of its different senses. In short, as the saying goes, not everything that can be (easily) counted counts, and vice versa.

Consequently, if we are to make progress in our understanding of children's acquisition of sentence-level constructions, we need to move away from models based only on surface form and towards models that include roles for abstract factors such as verb meaning, animacy, participant roles, construction-level semantics, and so on (e.g. St John & McClelland, 1990; Gordon & Dell, 2003; Chang *et al.*, 2006; Chang, 2009; Mayberry, Crocker & Knoeferle, 2009; see McCauley & Christiansen, 2014, for a review). Of course, if, as we have claimed, abstract frequency effects are important at the level of simple constructions, they are likely to be even more important when considering the more advanced constructions to which we now turn.

## MORE ADVANCED CONSTRUCTIONS

Both frequency effects in general, and our five theses in particular, scale up to more advanced constructions. Here we consider three construction types that have received considerable attention in the acquisition literature: questions (focusing mainly on *wh-*questions, which have tended to attract more research attention than *yes/no* questions), relative clauses, and passives.

### Questions

Most agree that the very first questions that English-speaking children produce are rote-learned, frequently heard, probably unanalyzed strings, such as *what's+that* (often pronounced as *whassat?*). Many would also agree with Klima and Bellugi (1966) that these very early questions include partially analyzed high-frequency formulae such as *What-X-(doing)?* and *Where-X-(going)?* (see also Fletcher, 1985). However, the role of frequency beyond these earliest formulaic utterances is more controversial. Here we argue that there is ample evidence that children's early question acquisition is moulded by input frequency well into development. We suggest that studies of question acquisition support three of our theses: (i) that frequent items are acquired before infrequent ones, all else being equal (AoA); (ii) that high-frequency question types can Prevent Errors; and (iii) under some circumstances, an over-reliance on high-frequency forms can Cause Errors).

First, studying the order in which children start to produce *wh*-words demonstrates that a word's frequency affects how easily and early it is acquired (AoA). *Wh-*questions in particular provide a good test bed for investigating the effect of frequency on the acquisition of lexical items because they contain a built-in control for many of the other variables that we know interact with (and can mask the effect of) frequency. For example, in English, *wh*-words always appear in the same position – at the beginning on the clause – so controlling for the effect of sentence position on an item's salience is not necessary. Similarly, all *wh*-words are roughly equivalent in ease of production since all are one-syllable words which start with one of two phonemes (/w/ for *what, where, why, when*, and *which* and /h/ for *how* and *who*).

A number of studies have observed a correlation between order of acquisition and input frequency in a range of languages. For example, Rowland, Pine, Lieven, and Theakston (2003) reported that the order in which the twelve Manchester corpus children began to produce English *wh-*words correlated with the frequency of the *wh-*words in their input, even when syntactic and semantic complexity were taken into account. Wode (1976), Forner (1979), Savic (1975), and Clancy (1989) have reported similar findings for German, Serbo-Croatian, and Korean (see also Tyack & Ingram, 1977; Bloom, Merkin & Wootten, 1982, for English; Okubo, 1967, for Japanese). Once again, input frequency is not the only relevant factor here, since it only accounted for only 13–36% of the variance in the order of *wh*-word acquisition (Rowland *et al.*, 2003), as predicted by our Interaction Thesis, but it is a significant factor nonetheless.

Research into children's questions (both *wh-* and *yes/no*) also demonstrates how highly frequent sequences can help protect children from making syntactic errors when constructing sentences (Prevent Error). Although word order errors are rare in children's early productions, English-learning children make a surprising number of these errors in their early question formation. These errors include subject–auxiliary inversion errors in which the tense- and agreement-marked auxiliary occurs post-, instead of pre-subject (e.g. *What he can do?) and double-marking errors in which tense+agreement is marked twice (*What did he didn't want; *What is he isn't eating?; *Does she doesn't want a drink?). These errors pattern systematically, and therefore cannot be dismissed as momentary lapses or slips of the tongue. For example, they are generally more common with some *wh-*words (e.g. *why*) and auxiliaries (e.g. DO and the modal auxiliaries), and with negative questions (e.g. *Why does she doesn't like it?; Can she can't see him?*; Ambridge, Rowland, Theakston & Tomasello, 2006; Rowland, 2007; Ambridge & Rowland, 2009; Rowland & Theakston, 2009).

The many different theoretical accounts of these errors that have been proposed need not concern us here (e.g. Stromswold, 1990; De Villiers, 1991; Valian, Lasser & Mandelbaum, 1992; Santelmann, Berk, Austin, Somashekar & Lust, 2002). The important point is that whatever other factors may affect rates of error (e.g. polarity and auxiliary type, as discussed above), questions are more susceptible to error when certain *wh-*words are combined with certain auxiliaries. For example, Rowland and Pine (2000) reported that one child, Adam, produced *Where shall* questions correctly but made errors with *What shall*. Similarly, he produced errors with *How can* but not with *How do.* These findings suggest that, whatever other rules or abstractions young children are using, they are making at least some use of high-frequency lexical frames learned from the input (e.g. *How do + X*; Rowland & Pine, 2000: Rowland, 2007; Ambridge & Rowland, 2009). The relevant questions are thus protected from error, since the word order of the question is specified directly in the frames.

If this is the case, then one would expect to see higher error rates for lower-frequency question types for which the child has no frame available, and must therefore be generated using other strategies (e.g. generalizing from existing knowledge). Rowland (2007; see also Dabrowska & Lieven, 2005; Ambridge & Rowland, 2009) directly tested the prediction that question types that had occurred with high frequency in the input would be picked up as frames by children and so would be protected from error. In an analysis of the *yes/no* and *wh*-questions produced by ten English-learning children aged two to five years, she reported significantly lower rates of error in question types that were highly frequent in the children's input than in low-frequency question types. Importantly, the analyses ruled out alternative explanations, such as the identity of the *wh*-word or auxiliary, or the input frequency of the individual words.

The domain of question acquisition also exhibits evidence for our Cause Error Thesis. An over-reliance on frequent frames can not only protect from error, but, in some cases, cause errors, when children use these frames inappropriately, for example by combining a *wh*-word+auxiliary frame (e.g. *Why can*), with an inappropriate declarative phrase (*she can't drink the milk*) to yield a doubling error (*Why can she can't drink it the milk?*; Dabrowska and Lieven, 2005, found that 20% of their potentially frame-derived questions were errors). Ambridge and Rowland (2009) tested this prediction in an elicitation experiment with English-learning three- to four-year-olds. They reported that doubling errors were more likely to be produced by children who had already learnt the relevant *wh*+auxiliary frame (*Why can*), and speculated that doubling errors occurred when children combined these frames with a declarative fragment (*Why can* + *she can't drink the milk*), suggesting that stored high-frequency strings can sometimes cause, as well as protect from, error.

Once again, this is a domain in which frequency interacts with other factors such as cognitive complexity (Interaction Thesis). For example, both Rowland (2007) and Ambridge and Rowland (2009) reported that certain question types (e.g. *Why don't*, and, indeed, most negative questions) attracted higher rates of error than would be expected solely on the basis of input frequency. Again, the conclusion that other factors are also at play does not obviate the need for a frequency-sensitive learning mechanism and, indeed, constrains theory development by highlighting the need for a mechanism that explains the interaction of frequency with other relevant factors.

Finally, it is important to note that an explanation of the frequency effects outlined in this section need not necessarily incorporate the assumption of item-based frames. For example, under Westergaard's (2009) approach, children are learning and applying grammatical movement rules (as in the generativist theories mentioned above), but these are framed in terms of language-specific micro-cues that specify in detail when and where different grammatical rules apply. Cues for which there is a lot of evidence in the input (i.e. high-frequency cues) will inevitably be learned first. Thus, as we argued in the ‘Introduction’, a frequency-sensitive account will not necessarily be a constructivist one; a point to which we return in the final section.

### Relative clauses

Throughout this article we have emphasized the existence of different types of frequency effect (Levels and Kinds Thesis), from those involving concrete strings to those involving abstract cues and constructions. In this section, we present evidence that frequency effects of the more abstract type are observed for children's acquisition of relative clauses. Thus, frequent forms, when appropriately defined, are associated with earlier acquisition (AoA) and lower error rates (Prevent Error).

At first glance, the bulk of past research on relative clauses (RCs) appears to present a clear counter-argument to the claim that frequency significantly influences acquisition. Most of this research has focused on the acquisition of subject (1) and object (2) RCs.

(1) The girl that chased the boy
(2) The boy that the girl chased

Let us first concentrate on the language for which we have the most data: English. Naturalistic and experimental studies suggest that children acquire subject RCs before object RCs (e.g. Diessel & Tomasello, 2000; Kidd & Bavin, 2002). Additionally, a host of adult sentence processing studies have consistently reported a subject advantage for RC processing (e.g. Gibson, 1998). These results, especially the experimental data, are consistent, and replicate across typologically similar languages. This pattern is problematic for any argument that frequency influences syntactic acquisition, since, in English, object RCs are more frequent than subject RCs in child-directed speech (Diessel, 2004) and in spoken language in general (Roland *et al.*, 2007). We argue in this section that, far from constituting evidence against a frequency-sensitive learning mechanism, the case of RCs reveals the multiplicity of levels in which frequency exerts an influence on acquisition (Levels and Kinds Thesis).

Subject and object RCs differ substantially in their functional-distributional properties. Fox and Thompson (1990) first identified a number of dimensions on which the two structures differ. One prominent dimension is the animacy of the head noun: subject RCs are significantly more likely than object RCs to contain an animate head noun, whereas the opposite is the case for inanimate heads. Second, object RCs typically contain discourse-old RC subjects. Finally, both Roland *et al.* (2007) and Fox and Thompson (2007) have shown that object RCs in spoken English rarely contain a relative pronoun. As such, although most experimental studies tested object RCs like (2), which contain two animate NPs and an overt relative pronoun, the types of object RCs that are most frequent in spoken discourse more closely resemble (3).

(3) The film I saw last night

The distributional tendencies of object RCs are attributable to two functional properties of language (Du Bois, 1987): (i) objects are typically inanimate, whereas subjects tend to be animate (typically human); and (ii) subjects tend to be discourse-old. These are statistical properties of language. The likelihood of overt relativizer (*that, which*) use is also subject to frequency constraints: Fox and Thompson (2007) identified several variables that predict the use/non-use of the relativizer, one being whether or not the RC subject was expressed as a pronoun (leading to non-use). Although these distributional facts are often ignored in studies of RC acquisition, they exert significant influences on children's acquisition.

Studies of naturalistic speech show that children quickly converge on these frequency patterns. Diessel (2009) reported on the distributional properties of subject and non-subject (predominantly object) RCs in Adam's (Brown, 1973) and Abe's (Kuczaj, 1976) speech from the CHILDES corpus (MacWhinney, 2000). Non-subject RCs overwhelmingly contained inanimate head nouns (91·7%) and pronominal RC subjects (88·1%) (see also Kidd, Brandt, Lieven & Tomasello, 2007). These numbers closely resembled the frequency of different NP-types in simple transitive clauses in the children's speech, where 86·9% of all subjects were first or second person pronouns. Therefore, despite the fact that non-subject RCs do not follow canonical word order, they do mark syntactic roles canonically (i.e. subject = animate, given, object = inanimate) and in a manner that matches the distributional properties of simple transitive sentences. Crucially, these frequency estimates from corpora predict children's correct production and comprehension of RCs in controlled experimental contexts. For instance, Kidd *et al.* (2007) and Brandt, Kidd, Lieven, and Tomasello (2009) showed that the typical subject–object asymmetry is neutralized and in some instances reversed when three- to four-year-old English- and German-speaking children were tested on highly frequent object RC types (i.e. those with an inanimate head noun and a pronominal RC subject) (see also Arnon, 2010).

Thus, as we saw in ‘Simple syntactic constructions’, children's acquisition of RCs is influenced by frequency, but at the level of abstract cues (e.g. animacy, givenness) and lexical items (i.e. pronouns) that are frequently associated with particular sentence positions. These distributional frequencies predict earlier acquisition (AoA), as well as lower error rates, and hence higher rates of correct performance, in both comprehension and production (Prevent Error).

Potentially problematic for this conclusion is the finding that subject RCs are actually the first type of RC to emerge in children's speech (Diessel & Tomasello, 2000). A closer inspection, however, reveals that the vast majority of these early RCs are so-called ‘presentational amalgam’ constructions, as in (4) and (5).

(4) Here's a mouse *go sleep*
(5) That is a train *go go*

Lambrecht (1988) described the presentational amalgam construction as a type of truncated RC, where the predicate nominal of the copular clause serves as the subject of the clause-final VP. Their status as true RCs in child language is equivocal: they are monoclausal and lack the obligatory relative pronoun. As such, they closely resemble canonical SV(O) clauses, leading to the possibility that children use their knowledge of frequent structural patterns to break into the syntax of RCs, after which their relative use of subject and object RCs closely approximates adult usage (see Fitz, Chang & Christiansen, 2011, for a connectionist model that uses word-order patterns learned from canonical SVO sentences to acquire the structure of relative clauses). Thus, again, we find that there are many different types of frequency effect (Levels and Kinds Thesis), and that, provided we define ‘form’ at the appropriate level, more frequent forms are associated with earlier acquisition (AoA Thesis).

One final emerging piece of evidence regarding the role of frequency in RC acquisition comes from languages other than English. Several researchers have suggested that the traditional subject–object asymmetry observed in experimental studies of English (and other typologically similar languages) derives from the fact that subject RCs follow canonical word order, whereas object RCs do not (e.g. Bever, 1970; MacDonald & Christiansen, 2002). This account makes the following prediction: object RCs should be acquired first and should be easier to understand in languages where their word order follows canonical word order. Chinese languages such as Mandarin and Cantonese follow this pattern. Although there are many more studies to conduct on these languages, there is some evidence in support of this prediction (Yip & Matthews, 2007; Chan, Matthews & Yip, 2011; Chen & Shirai, 2014; though see Hsu, Hermon & Zukowski, 2009). Thus, again, we see an effect of frequency, but at a very abstract level: the frequency of particular orderings of SUBJECT and OBJECT roles in the language as a whole; an effect far removed from a view under which the acquisition mechanism is sensitive only to the frequency of particular surface strings.

Whilst the evidence for frequency effects in this domain is clear, what remains unclear is how these effects are represented and implemented on-line. For instance, there is some evidence to suggest that many object RCs are produced using prefabricated chunks (e.g. *the one pro VERB*; see Fox & Thompson, 2007; Reali & Christiansen, 2005), but the processing advantage shown for object RCs that have less prototypical features (e.g. *the pen that I bought*) raises the possibility that the constraints of animacy and RC subject might be implemented incrementally on-line (see Kidd *et al.*, 2007). Given the importance of the wider question of the locus of frequency effects observed in first language acquisition, this is clearly an issue that requires further investigation.

### Passives

Research on passives illustrates that frequency effects can be found not only within a given language, but also cross-linguistically (Levels and Kinds Thesis): across languages, a negative correlation is often observed between the relative frequency of a particular construction in the language and the age at which it is typically acquired by its speakers (AoA Thesis). Passives are highly dispreferred in languages like English, German, and Hebrew, and thus occur infrequently. Our most comprehensive naturalistic data come from English: in a large corpus study, Xiao, McEnery, and Qian (2006) reported that the percentage of all passive types (full and truncated, using either *be* or *get*) in spoken British English is 0·16%. Using the Brown (1973) corpus (i.e. American English), Gordon and Chafetz (1991) reported that full passives occur in only ·005% of all sentences in CDS, whereas truncated passives occur 0·1% of the time. Not surprisingly, passives are also rare in the spontaneous speech of English-speaking children (Pinker, Lebeaux & Frost, 1987; Israel, Johnson & Brooks, 2000), a finding that is similar to reports on German (Mills, 1985) and Hebrew (Berman, 1985).

The learnability problem posed by infrequent and more advanced structures is well- worn territory in child language research, and the passive has been central to this debate. One way to evaluate how frequency matters is to compare languages such as English and German, in which the passive is infrequent, to languages where the passive occurs with much higher frequency. Indeed, there are several cases in the literature where higher passive frequency results in earlier acquisition (AoA Thesis). For instance, in Sesotho the passive is estimated to be ten times more frequent than it is in English (Kline & Demuth, 2010), which appears to result in comparatively earlier acquisition (Demuth, 1989; Demuth, Moloi & Machobane, 2010). Similar effects have been reported for Inuktitut (Allen & Crago, 1996), Bahasa Indonesia (Gil, 2006), and Ki'che’ Maya (Pye & Quixtan Poz, 1988). In every case the high frequency of passive use appears to stem from particular typological properties of the languages, which, in comparison to European languages, make the passive a less marked structure (Interaction Thesis).

Training studies in English complement the cross-linguistic work. In an early study, Whitehurst, Ironsmith, and Goldfein (1974) showed that modelling passives to four- to five-year-olds increased their production and comprehension, a finding corroborated by Vasilyeva, Huttenclocher, and Waterfall (2006) (for a training study of rare subject RCs in Turkish, see Sarilar, Matthews & Küntay, 2013). The Whitehurst *et al.*, study predates the structural priming literature (e.g. Bock, 1986; Pickering & Ferreira, 2008), but nowadays would be interpreted as a priming effect. The passive is the most studied structure in priming studies conducted with developmental populations, showing a consistent priming effect (e.g. Savage, Lieven, Theakston & Tomasello, 2003; Huttenlocher, Vasilyeva & Shimpi, 2004; Messenger, Branigan & McLean, 2011; Kidd, 2012).

The robust nature of the priming effect for the English passive has been explained with reference to the structure's low frequency – the so called inverse frequency effect, which describes the tendency for low-frequency structures to yield higher priming effects. Several explanations for this inverse frequency effect have been proposed, but the one that most naturally extends to acquisition is the argument that structural priming effects reflect implicit learning of structure (Chang *et al.*, 2006): children have a greater tendency to produce low-frequency forms after being primed because priming leads to larger representational change in comparison to more entrenched structures (e.g. the active transitive). Importantly, the account predicts that children will respond to low-frequency forms such as the passive differently across development: representational change in young children following exposure will be greater than in older children (effectively, younger children have more to learn). This leads to a prediction (or even perhaps a caution): we should not expect frequency effects to be uniform across developmental stages and, indeed, individual children (Levels and Kinds Thesis).

Finally, the acquisition of the passive has been shown to be either supported or hindered by its similarity or dissimilarity to other structural patterns. Abbot-Smith and Behrens (2006) showed that a German-speaking child acquired the stative *sein*-passive before the eventive *werden*-passive, even though the two forms are roughly equal in frequency in the input. However, the two passives overlap with other structures that serve to either support (in the case of the *sein*-passive) or hinder acquisition (in the case of the *werden*-passive). The acquisition of the *sein*-passive is facilitated by the previously learned morphologically and functionally similar present perfect, whereas the *werden*-passive cannot build on a previously acquired construction and competes in function with high-frequency modal verb constructions. Thus we have another instance where frequency at multiple levels interacts with other properties of language, in this case structural overlap, to determine acquisition (Interaction Thesis).

To conclude this section, there is ample evidence to suggest that frequency effects are observed not only for lexical strings and simple structures, but also for more advanced structures including questions, relative clauses, and passives. Because, in many cases, these frequency effects occur at the level of abstract categories, patterns, or cues, they are often more difficult to detect than frequency effects at the single-word or even construction level. When the data are analyzed at the appropriate level of abstraction, however, we see exactly the same types of frequency effect that are observed for other domains. One pressing challenge for future research in this domain is to better determine how frequency effects interact with other features of language, such as typology (e.g. see papers in Kidd, 2011).

## THEORETICAL IMPLICATIONS

The present article reviewed frequency effects in four core domains: the acquisition of single words, inflectional morphology, simple syntactic constructions, and more advanced constructions. We argued that frequency effects are ubiquitous across all of these domains, and, indeed, across language acquisition in general. In summarizing this evidence, we argued that there exist different types of frequency effect; for example, effects at the levels of lexical strings and abstract sentence constructions, as well as effects of both type and token frequency and of relative and absolute frequency (Levels and Kinds Thesis). We presented evidence that high-frequency forms are associated with earlier acquisition (AoA Thesis) and lower rates of error (Prevent Error Thesis), but also that they can cause error when used inappropriately (Cause Error Thesis). Finally we argued that frequency effects interact with other effects, such as utterance position, and that such interactions can be informative with regard to the nature of the language acquisition mechanism (Interaction Thesis).

Whether or not we have succeeded in convincing the reader of all of these individual claims, we hope to have marshalled sufficient evidence to convince all but the most hardened classicist (in the sense of Newmeyer, 2003) of the ubiquity of frequency effects across all domains of child language acquisition, and that frequency effects therefore constitute a phenomenon for which any successful theory must be able to account.

As we noted in the ‘Introduction’, this might be either a generativist/nativist account that assumes knowledge of innate syntactic categories, principles, and parameters (e.g. Yang, 2004; Westergaard, 2009) or a constructivist/usage-based account that does not (e.g. Tomasello, 2003). In principle, both classes of account could, given certain assumptions, explain the patterns of frequency effects outlined here. This is not to say, however, that all current theories can explain frequency effects, and that, by making reference to accounts that are incompatible with such effects, we are setting up a straw man. We have already mentioned in passing one account that explicitly denies any meaningful effect of frequency (Roeper, 2007). Much more common are proposals that do not explicitly rule out frequency effects (or, indeed, discuss them at all), but that posit learning procedures that not only (a) yield no frequency effects in their current form, but also (b) could yield no frequency effects without abandoning the core learning mechanism assumed.

An example is the triggering approach to setting word order parameters. Under such accounts (e.g. Sakas and Fodor, 2012), children acquire the word order of their language (e.g. SVO for English), not by abstracting across input utterances, but by setting syntactic parameters (e.g. setting the specifier–head and head–complement parameters to the settings that yield SV and VO, respectively). Because the account includes no role for input-based learning, it does not explain the finding that word order is better learned for more frequent verbs (Matthews *et al.*, 2005, 2007). Neither can the account straightforwardly be modified to yield such effects. It would be necessary to add the assumption that children learn word order by abstracting across input strings, which entirely obviates the need for the parameter-setting mechanism. The whole point of the account is to explain how children could use triggers to acquire word order rapidly, without having to build this knowledge gradually on the basis of the input. Thus there exist at least some accounts with which the type of frequency effects discussed in the present article are incompatible in principle.

However, while some individual accounts are incompatible with frequency effects, this is not true for whole families of accounts. Both constructivist and generativist accounts (including some parameter-setting accounts) can incorporate frequency-sensitive learning mechanisms. That said, we feel that it would be remiss of us to end this review sitting on the fence, and that we owe it to readers who have persisted this far to nail our colours to the theoretical mast. It will come as no surprise to anyone who has read any of our previous papers that these colours are those of the constructivist camp. But this is not a matter of research tradition, terminology, or simple preference; on our view, the constructivist account offers a more parsimonious account of frequency effects.

Let us illustrate this claim by returning to one of the domains that we have discussed here – inflectional morphology – and, specifically, to a phenomenon to which we have already alluded briefly. The phenomenon is that children sometimes produce agreement-/tense-less verb forms in contexts in which an inflected (here third person singular *-s*) form is required (e.g. *Dolly eat it). Importantly, both sides agree that this phenomenon is related to the input. For example, English and Dutch children hear these agreement-/tense-less verb forms frequently (e.g. in sentences such as *Let Dolly eat it* and *Dolly can eat it*), and so produce these errors at high rates. Italian and Spanish children hear these forms much less frequently, and so produce these errors rarely. Thus both generativist and constructivist researchers agree that this phenomenon can be explained only by positing some kind of frequency-sensitive learning mechanism.

Under a generativist account (e.g. Legate & Yang, 2007), children use the input to set an innately given TENSE parameter to either a positive (the language requires tense/agreement marking) or negative setting (it does not). Because this parameter is set probabilistically on the basis of the input – i.e. in a way that is frequency sensitive – this account can explain why English and Dutch children, who hear these ‘bare’ forms frequently, produce more errors that Italian and Spanish children, who do not.

Under the constructivist account (e.g. Freudenthal *et al.*, 2007; Räsänen *et al.*, 2014) children make these errors because they are learning from the input individual lexical forms and multiword strings (e.g. *play, plays, Let Dolly play*, etc.), which they sometimes use inappropriately (e.g. producing *Let Dolly play*, in a context where *Dolly plays* would be appropriate). This proposal not only offers a closer fit to the quantitative cross-linguistic pattern, but also explains why – within a given language – some verbs display higher error rates than others (Freudenthal *et al.*, 2010). For example, in English, the verbs that children frequently hear in ‘bare’ versus third person singular *-s* form, particularly in utterance-final position, are exactly those verbs that children frequently produce in bare form in third singular contexts (Theakston, Lieven & Tomasello, 2003; Kirjavainen, Theakston & Lieven, 2009; Freudenthal *et al.*, 2010; Räsänen *et al.*, 2014).

Now, as we argued above, there is no reason in principle why the generativist account could not be adapted to accommodate these lexical-level frequency findings. One could quite easily propose that, in addition to using input forms to set the TENSE parameter (Legate & Yang, 2007), children additionally store input strings and, on a non-negligible proportion of occasions, produce utterances by retrieving these stored strings directly. Why then, do we favour the constructivist alternative? The reason is that the constructivist account yields these lexical input frequency effects naturally, using the core learning mechanism assumed by the account (i.e. the storage and reuse of strings from the input). In contrast, the generativist account yields these effects by discarding the core mechanism assumed by that account (at least, on a sufficiently large proportion of occasions for the effects to be detectable) and adding ancillary hypotheses that have no independent theoretical motivation within the account; that serve no purpose other than to explain otherwise recalcitrant findings.

An analogous situation applies in every domain that we have investigated. For example, children could acquire word order by setting innate complement–head and specifier–head parameters that spell out (amongst other things) the target order of the innate categories of SUBJECT, VERB, and OBJECT in the language being learned. But in order to explain the finding that children and adults have detailed knowledge of the frequency with which particular verbs have appeared in this construction, the generativist account would have to add the assumption that – in addition to setting this parameter – children record verb+construction collocation frequencies. Again, whilst for the generativist account this assumption is merely an ancillary hypothesis with no independent theoretical motivation, the phenomenon falls naturally and inevitably out of the constructivist account: if children learn the SUBJECT VERB OBJECT construction by abstracting across particular instances of that construction in the input, then the frequency with which each verb has appeared in this construction is immanent in the generalization. We would be the first to admit that there are many important language acquisition phenomena for which current constructivist accounts do not offer a satisfactory explanation; but, on our view, constructivist accounts, which have frequency sensitivity built into their very fabric, provide the most parsimonious explanation of the multiplicity of frequency effects discussed here.

To summarize, the current article has presented evidence of pervasive frequency effects across children's language acquisition. Frequency effects are observed across a variety of different domains, levels (e.g. lexical vs. abstract; type vs. token, absolute vs. relative), and outcome measures (e.g. age of acquisition, rates of error/correct use, types of error), and therefore constitute a phenomenon that demands explanation under any theoretical account. Although we have advocated a constructivist account, this is not to say that alternative approaches are incompatible with frequency effects in principle. The challenge for such accounts is to incorporate motivated mechanisms that yield frequency effects whilst preserving the core mechanistic assumptions of the account.

In conclusion, whilst – as we have tried to stress throughout – frequency isn't everything, frequency certainly isn't nothing. On the contrary, frequency effects constitute a phenomenon that any successful account of child language acquisition must explain.
